# 2,5-Hexanedione induces dopaminergic neurodegeneration through integrin α_M_β2/NADPH oxidase axis-mediated microglial activation

**DOI:** 10.1038/s41419-017-0091-7

**Published:** 2018-01-19

**Authors:** Cong Zhang, Liyan Hou, Jie Yang, Yuning Che, Fuqiang Sun, Huihua Li, Qingshan Wang

**Affiliations:** 1 School of Public Health, Dalian Medical University, No. 9W. Lvshun South Road, Dalian, 116044 China; 2 Department of Cardiology, Institute of Cardiovascular Diseases, First Affiliated Hospital of Dalian Medical University, Dalian, China

## Abstract

Recent study demonstrated that chronic exposure to solvents increases the risk of Parkinson’s disease (PD), the second most common neurodegenerative disorder characterized by progressive dopaminergic neurodegeneration in the substantia nigra (SN). *n*-Hexane, a widely used organic solvent, displays central-peripheral neurotoxicity, which is mainly mediated by its active metabolite, 2,5-hexanedione (HD). However, whether HD exposure contributes to PD remains unclear. In this study, we found that rats exposed to HD displayed progressive dopaminergic neurodegeneration in the nigrostriatal system. Microglial activation was also detected in HD-treated rats, which occurred prior to degeneration of dopaminergic neurons. Moreover, depletion of microglia markedly reduced HD-induced dopaminergic neurotoxicity. Mechanistic study revealed an essential role of microglial integrin α_M_β_2_-NADPH oxidase (NOX2) axis in HD-elicited neurotoxicity. HD activated NOX2 by inducing membrane translocation of NOX2 cytosolic subunit, p47^phox^. Integrin α_M_β_2_ was critical for HD-induced NOX2 activation since inhibition or genetic deletion of α_M_β_2_ attenuated NOX2-generated superoxide and p47^phox^ membrane translocation in response to HD. Src and Erk, two downstream signals of α_M_β_2_, were recognized to bridge HD/α_M_β_2_-mediated NOX2 activation. Finally, pharmacological inhibition of α_M_β_2_-NOX2 axis attenuated HD-induced microglial activation and dopaminergic neurodegeneration. Our findings revealed that HD exposure damaged nigrostriatal dopaminergic system through α_M_β_2_-NOX2 axis-mediated microglial activation, providing, for the first time, experimental evidence for *n*-hexane exposure contributing to the etiology of PD.

## Introduction

Parkinson’s disease (PD) is a common neurodegenerative disorder, generally affecting ~2% of the population over 65 years old. Clinically, PD is mainly characterized by movement disorder including rigidity, resting tremor, bradykinesia, and postural instability, which is mainly due to the loss of dopaminergic neurons in the substantia nigra pars compacta (SNpc)^1,2^. Current dopamine replacement therapy for PD only provides relief of motor symptoms and fails to interrupt the progressive neurodegenerative process. Therefore, exploring the etiology of PD is urgently needed for the prevention of this disorder.

Accumulating evidence suggests that both genetic mutation and exposure to environment factors contribute to the onset and development of PD. Although a number of genetic mutations are found in PD patients, most PD cases (~90%) are sporadic^3^. Furthermore, the low rate of concordance for PD in monozygotic and dizygotic twins^4,5^ strongly supports that environmental factors are critical for acquiring PD. Epidemiological and case-control studies suggest that exposure to environmental toxicants, such as pesticides, heavy metals and solvents increases the risk of PD^6^. Although solvents have long been considered as a potential risk factor of PD, they gain less attention than pesticides or heavy metals despite their widespread usage in industry^7^. Some of solvents are lipophilic and are easily absorbed by central nervous system (CNS)^8^, resulting in neurological damage^9,10^. There are isolated cases of acute Parkinsonism associated with solvents exposure, such as in workers exposed to *n*-hexane^10,11^ or toluene^12^. However, there is still lacking experimental evidence for the involvement of solvents in promoting the degeneration of the nigrostriatal dopaminergic systems.

*n*-Hexane, an organic solvent, is widely used in various industrial processes, such as paints, varnishes, printing inks and shoe manufacturing. As a common environmental contaminant, a small amount of *n*-hexane and its toxic metabolite, 2,5-hexanedione (HD), can be detected in urine samples of human, which is greatly elevated in workers exposed to *n*-hexane^13,14^. The peripheral neurotoxicity of *n*-hexane and HD is well-documented in previous studies; however, the research on the toxic effects of *n*-hexane on dopaminergic neurons in the CNS and related mechanisms is very limited. PD patients display reduced capacity of *n*-hexane elimination^15^. Moreover, parkinson-like symptoms are observed in human with long-term occupational exposure of *n*-hexane^16^. Positron emission tomography study reveals regional striatal abnormality of the dopaminergic system in *n*-hexane-exposed patients^17^. Furthermore, experimental animals treated with HD exhibit high levels of apomorphine-induced rotational behavior compared to vehicle controls^18^. We therefore hypothesized that exposed to *n*-hexane might be toxic to dopaminergic neurons, contributing to the onset of PD.

To test our hypothesis, adult SD rats were treated with HD and the loss of nigrostriatal dopaminergic neurons as well as the underlying mechanisms in HD-intoxicated rats was investigated in the present study.

## Results

### HD induces dopaminergic neurodegeneration and α-synuclein oligomerization

**Fig. 1:**
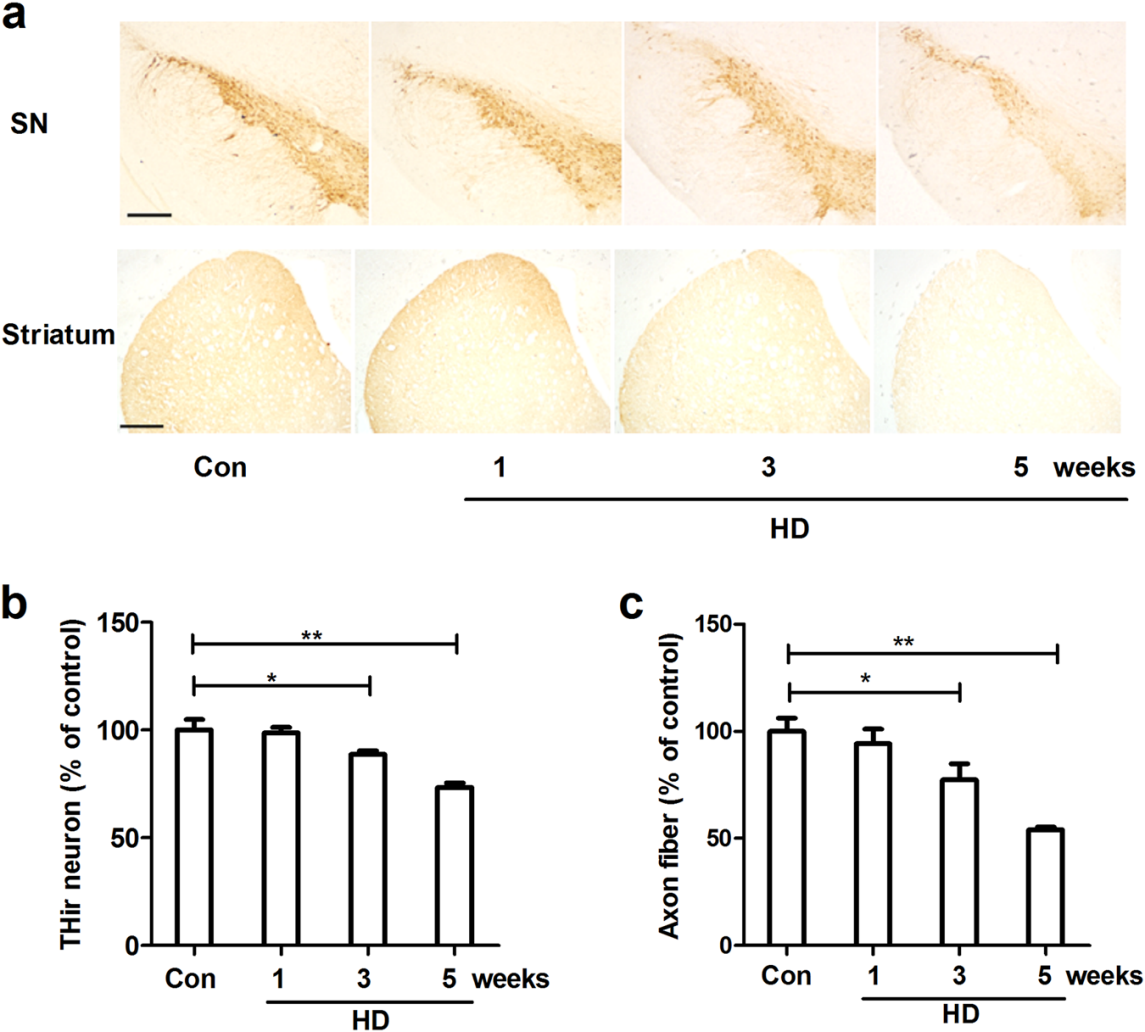
HD exposure induces progressive dopaminergic neurodegeneration **a** After 1, 3, and 5 weeks of HD treatment, dopaminergic neurons in the SN and striatum were immunostained with antibody against TH and the representative images were shown. **b** The number of TH^+^ neurons in the SNpc was counted. **c** Striatal dopaminergic neuron fibers were quantified by TH density. *p < 0.05, **p < 0.01; *n* = 4–6; Scale bar = 200 μm

To determine whether HD damages dopaminergic neurons, HD was administrated to rats. The dopaminergic neurons in the SNpc were identified by anti-TH immunostaining and the number of TH-immunoreactive (THir) cells was counted at different time points of HD intoxication. As illustrated in Fig. 1a, compared with vehicle controls, HD-treated rats displayed significant loss of THir cells in the SNpc in a time-dependent manner. Quantitative analysis showed that exposure to HD resulted in 12.29% (±2.81%, *F*_(3,14)_ = 16.846, *p* < 0.05) and 27.70% (±3.59%, *F*_(3,14)_ = 16.846, *p* < 0.001) loss of nigral dopaminergic neurons after 3 and 5 weeks of treatment, respectively (Fig. 1a). In addition to loss of cell body, the axon fibers of dopaminergic neurons in the striatum were also damaged by HD as shown by a reduced level of TH density compared to vehicle controls (Fig. 1a and c).

### Microglial activation precedes dopaminergic neurodegeneration in HD-treated rats

**Fig. 2:**
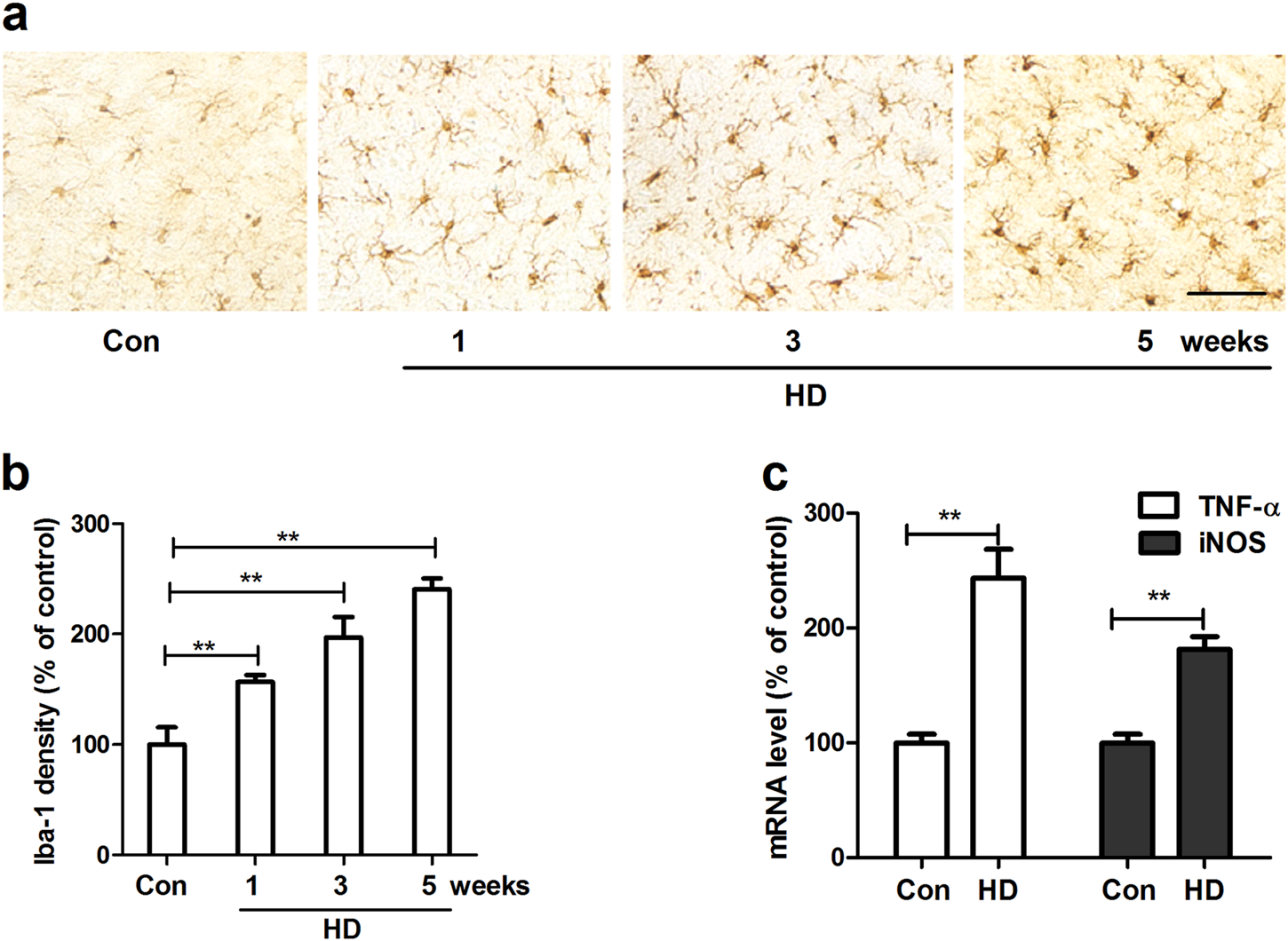
HD exposure induces microglial activation in the SN of rats **a** After 1, 3, and 5 weeks of HD treatment, microglia in the SN were stained with antibody against Iba-1 and the representative images were shown. Activated microglia display larger cell body size and intensified Iba-1 staining. **b** Microglial activation was quantified by calculating the density Iba-1 immunostaining. **c** The gene expressions of TNF-α and iNOS were measured in the midbrain of HD-treated rats by using RT-PCR. *p < 0.05, **p < 0.01; *n* = 4–6; Scale bar = 50 μm

Neuroinflammation mediated by microglia, the innate immune cells in the brain, plays a critical role in the progressive dopaminergic degeneration in PD. We therefore determined the effects of HD on microglial activation. Microglia in the SN were stained with antibody against Iba-1, a microglial marker. As shown in Fig. 2a, compared with vehicle controls, microglia in HD-treated rats displayed hypertrophied morphology and intensified Iba-1 staining after 1 week of HD exposure, indicating microglial activation. Activated microglia in the SN were continually observed up to 5 weeks after HD intoxication. Quantitative analysis supported the morphological observation by showing 156.50% ± 12.85%, 212.48% ± 25.51%, and 249.73% ± 20.26% increase of Iba-1 density in SN of rats after 1, 3, and 5 weeks of HD exposure, respectively, compared with vehicle controls (Fig. 2b).

Activated microglia produce a wide array of inflammatory cytokines, such as tumor necrosis factor-α (TNF-α) and nitric oxide (NO), that act on neurons to induce damage. The gene expressions of TNF-α and inducible NO synthase (iNOS) were detected in the midbrain of rats after 5 weeks of HD intoxication. In agreement with microglial activation, HD exposure significantly elevated the mRNA levels of TNF-α and iNOS (Fig. 2c).

### Microglia are essential for HD-induced dopaminergic neurodegeneration

**Fig. 3:**
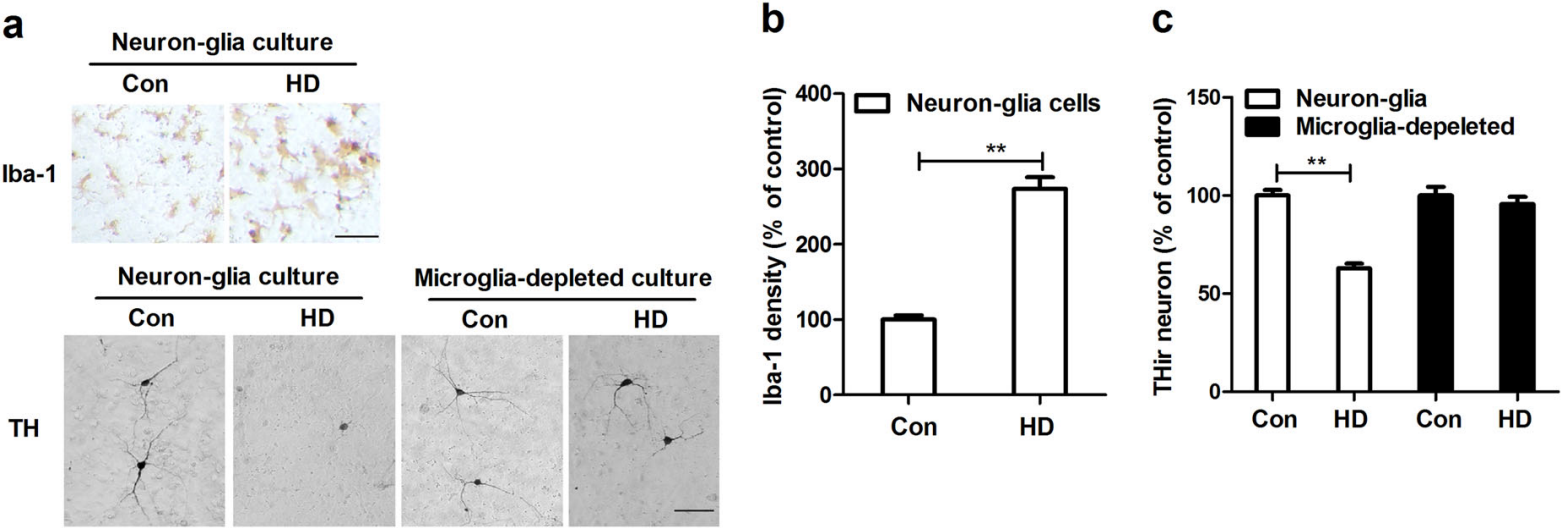
Microglia are essential for HD-induced dopaminergic neurodegeneration **a** Rat primary midbrain neuron–glia or microglia-deleted cultures were stained with antibody against Iba-1 or TH after 2 days of HD treatment and the representative images were shown. **b** The density of Iba-1 staining was quantified. **c** The number of TH^+^ neurons was counted in both neuron–glia and microglia-deleted cultures. Results were expressed as a percentage of controls from three experiments performed in duplicate.*p < 0.05, **p < 0.01; Scale bar = 50 μm

To dissect the mechanism underlying HD-induced dopaminergic neurodegeneration, primary midbrain neuron–glia cultures were prepared and treated with 8 mM HD. The chosen concentration of HD was based on our previous report^19^. Consistent with in vivo, HD treatment induced activation of microglia in neuron–glia cultures, which was accompanied with 33.93% ± 5.17% (*t* = 4.867, *p* < 0.05) loss of dopaminergic neurons (Fig. 3a–c). In addition to loss of cell body, dopaminergic neurons in HD-treated cultures also displayed a less extensive dendritic network compared with those in controls (Fig. 3a).

To determine whether microglial activation contributes to HD-induced dopaminergic neurotoxicity, microglia were removed from midbrain neuron–glia cultures (containing ~10% microglia) by l-leucine methyl ester (LME). We recently reported that, in our conditions, LME is capable of depleting more than 99.9% microglia and displays no significant effects on survival of dopaminergic neurons and functions of astrocyte^20^. Microglia-depleted cultures were compared with the neuron–glia cultures for their susceptibility to HD-induced dopaminergic neurotoxicity. As seen in Fig. 3a and c, unlike the neuron–glia cultures, HD-induced dopaminergic neurodegeneration was abolished in microglia-depleted cultures. This finding suggests that HD-induced dopaminergic neurotoxicity required the presence of microglia, most likely through dys-regulated microglial activation.

### HD exposure induces activation of NADPH oxidase

**Fig. 4:**
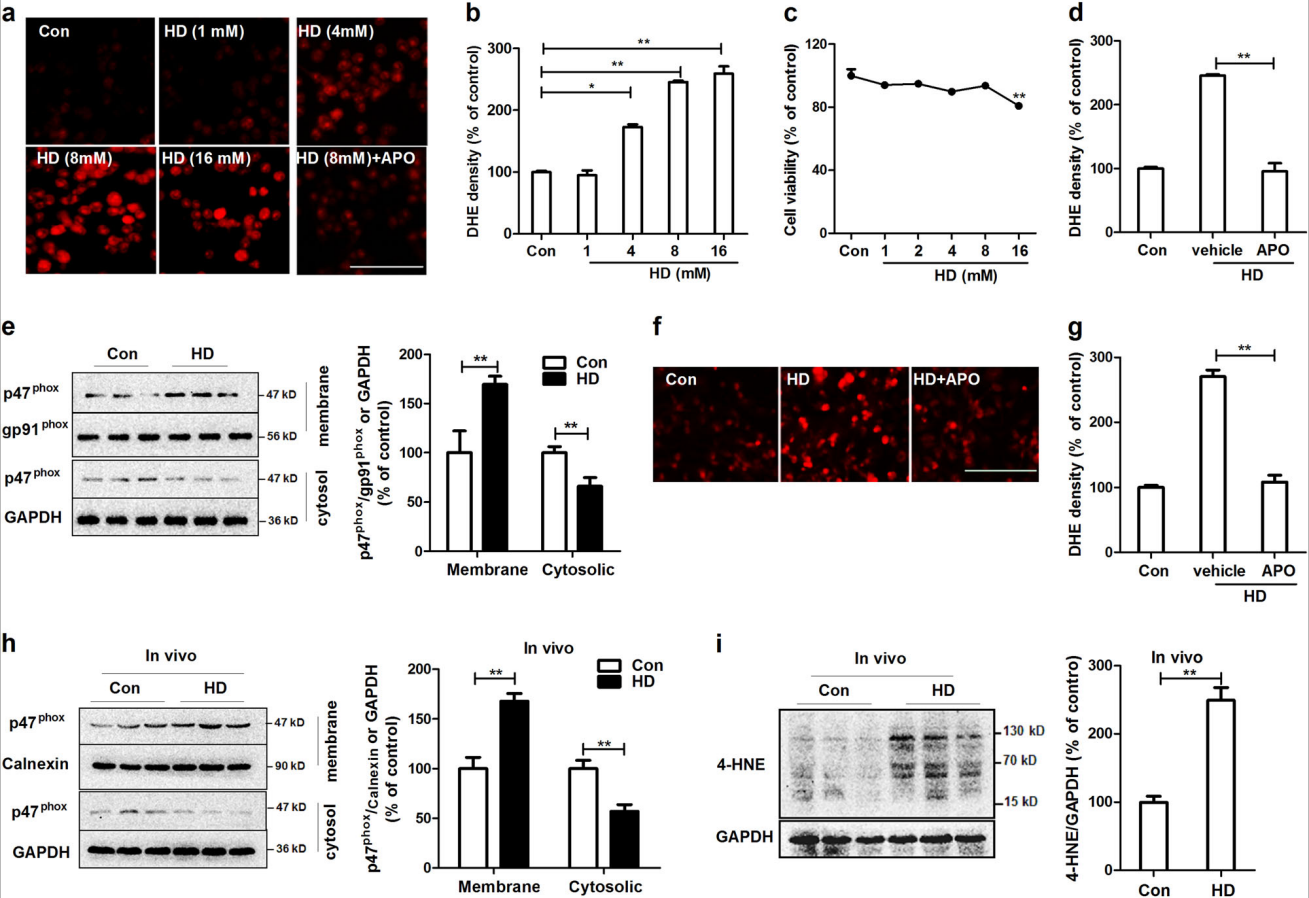
HD activates NOX2 **a** BV2 microglial cells were treated with 1, 4, 8, and 16 mM HD with or without apocynin (APO) pre-treatment. The production of superoxide was assessed by DHE and the representative images of DHE oxidation were shown. **b** The density of red fluorescence of DHE oxidation was quantified. **c** Cell viability of BV2 microglia treated with different concentrations of HD was detected using LDH assay. **d** The effects of apocynin on HD-induced superoxide production was quantified. **e** The membrane translocation of NOX2 cytosolic subunit, p47^phox^ in HD-treated BV2 microglial cells was detected after 30 min of HD stimulation by using Western blot and the density of blots was quantified. Gp91phox, an abundant membrane protein, and GAPDH were used as an internal membrane and cytosolic control, respectively. Previous reports indicated that gp91phox is a reliable internal membrane control^52,60^. **f** HD-induced superoxide production was examined in primary mixed-glia cells by DHE and the representative images of DHE oxidation were shown. **g** The density of red fluorescence of DHE oxidation was quantified. Results were expressed as a percentage of controls from three experiments performed in duplicate. **h** The membrane translocation p47^phox^ was detected in midbrain tissues of HD-treated rats by Western blot and the density of blots was quantified. **i** The levels of 4-HNE were determined in midbrains of HD-treated rats by Western blot and the density of blots was quantified. *p < 0.05, **p < 0.01; *n* = 3–6; Scale bar = 100 μm

We recently demonstrated that NADPH oxidase (NOX2), a superoxide-producing enzyme, plays an important role in the initiation and maintenance of microglial activation and related neurodegeneration in multiple models of PD^21^. To determine whether NOX2 is involved in HD-induced microglial activation and dopaminergic neurodegeneration, the effects of HD on NOX2 activation were initially investigated. NOX2 is a multimeric enzyme composed of membrane (gp91^phox^, p22^phox^) and cytosolic (p47^phox^, p67^phox^, p40^phox^ and Rac1/2) subunits. Membrane translocation of cytosolic subunits is an essential step for NOX2 activation^22–24^. Therefore, NOX2 activation was examined by using both production of superoxide and membrane translocation of NOX2 cytosolic subunit. Primarily microglia are known to be short lived and become activated in the condition without the presence of neuron or astrocyte, BV2 microglial cells were therefore initially used. Previous study demonstrated that BV2 cell is an ideal alterative model for primary microglia for examining brain inflammation^25^. In situ visualization of superoxide production was performed using DHE, a ROS-sensitive dye that exhibits red fluorescence through interactions with superoxide. As shown in Fig. 4a and b, superoxide production in vehicle-treated microglial cells was minimal as evidenced by the low level of red fluorescence. In contrast, exposure to 4, 8, and 16 mM HD resulted in gradual increase of red fluorescence, indicating elevated ROS production. To rule out the possibility that the increase of ROS production was attributed to nonspecific toxicity of HD, we evaluated effect of various concentrations of HD on the viability of microglial cells. HD at concentrations of 8 mM and lower did not show significant toxicity (Fig. 4c). Furthermore, HD (8 mM)-induced increase of superoxide production was significantly suppressed by apocynin (APO), a widely used NOX2 inhibitor (Fig. 4d), suggesting that NOX2 accounts for the major source of HD-induced superoxide. In agreement with elevated superoxide, HD exposure also induced membrane translocation of NOX2 cysotolic subunit, p47^phox^. Western blot analysis showed that compared with vehicle controls, the levels of p47^phox^ in the membrane fractions of HD-treated microglia were significantly increased, and meanwhile, were significantly decreased in cytosolic fractions (Fig. 4e), indicating NOX2 activation.

To further support this conclusion, primary mixed-glia cultures that are containing ~20% microglia were prepared and treated with HD. The reasons for using mixed-glia cells instead of purified microglia are as follows: (1) primarily microglia are short-lived and become activated in the condition without the presence of either neurons or astrocytes; (2) it is difficult to harvest a sufficient amount of purified microglia for experiments; (3) we previously reported that microglial NOX2 is the major source of superoxide production in mixed glial culture^26,27^. Consistently, a higher level of superoxide was observed in primary mixed-glia cells treated with HD than vehicle controls, which was significantly suppressed by NOX2 inhibitor apocynin (Fig. 4f and g). Due to the consistency for superoxide production and p47^phox^ membrane translocation to reflect NOX2 activation, the membrane translocation of p47^phox^ in HD-treated primary microglia was not examined.

To determine whether HD-induced NOX2 activation occurred in vivo, p47^phox^ membrane translocation was examined in HD-treated rats. Consistent with in vitro, HD significantly increased the levels of p47^phox^ in the membrane factions of midbrain tissues compared with vehicle controls (Fig. 4h). Coincidentally, a reduced level of p47^phox^ in the cytosol of midbrains prepared from HD-treated rats was observed (Fig. 4h). In agreement with NOX2 activation, the levels of 4-HNE, a mediator and product of oxidative stress, was also increased in the midbrains of HD-treated rats compared with vehicle controls (Fig. 4i).

### Integrin α_M_β_2_ but not scavenger receptors is involved in HD-induced NOX2 activation

**Fig. 5:**
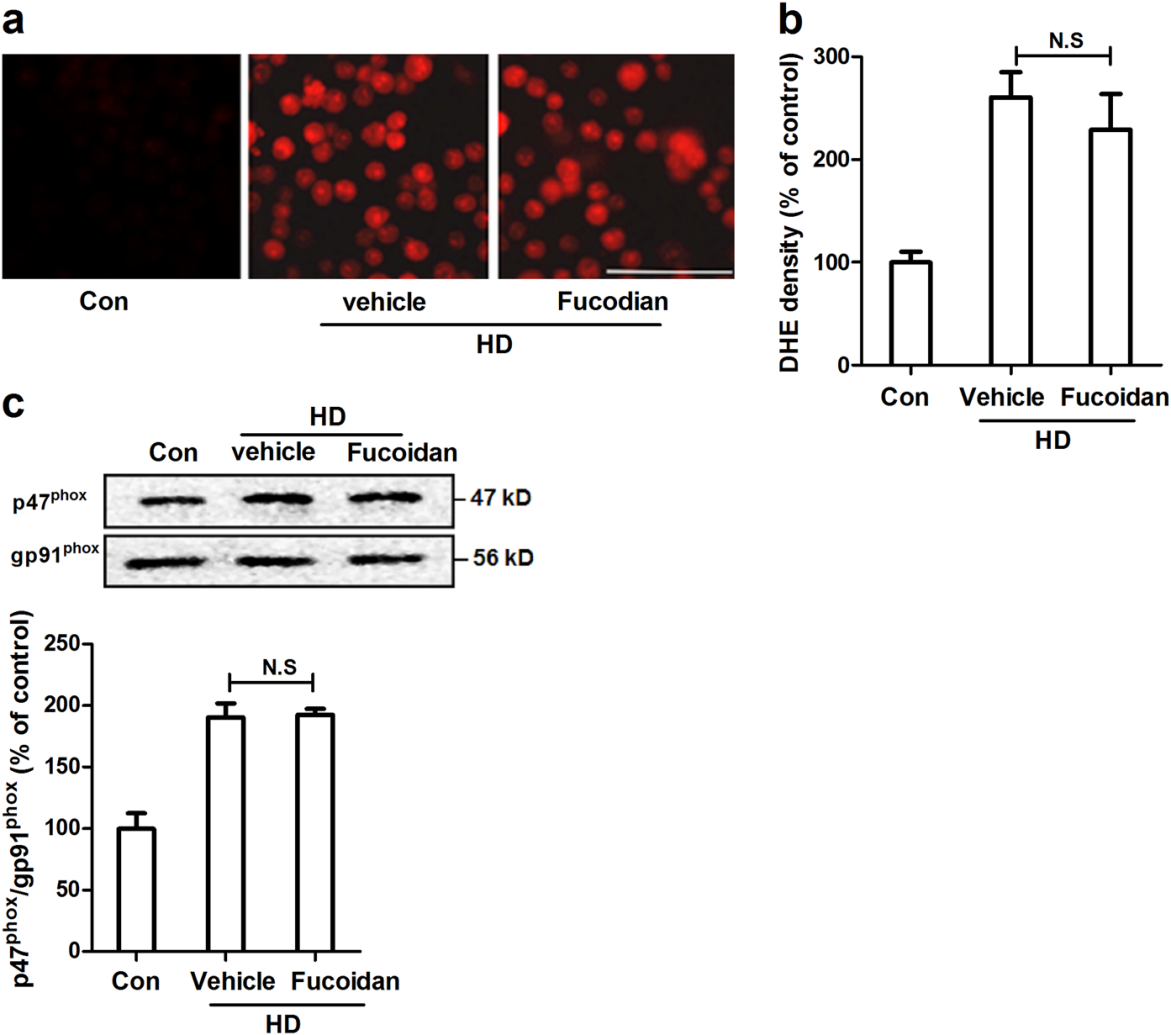
SRs inhibitor fucoidan fails to block HD-induced NOX2 activation **a** The production of superoxide induced by HD was assessed in BV2 microglial cells with or without fucoidan pre-treatment. **b** The density of red fluorescence of DHE oxidation was quantified. **c** The effects of fucoidan on HD-induced p47^phox^ membrane translocation was detected using Western blot and the density of blots was quantified. Results were expressed as a percentage of controls from three experiments performed in duplicate. N.S. = not significant. Bar = 100 μm

Previous studies indicated that pattern recognition receptors (PRRs) are essential for microglial activation and reactive oxygen species (ROS) production in response to damage associated molecular patterns^28^. Scavenger receptors (SRs) and integrin α_M_β_2_ (also called CD11b/CD18 or Mac1) are two members of PRRs family and are highly expressed in microglia. We therefore investigated the role of SRs and α_M_β_2_ in HD-induced NOX2 activation. Fucoidan, a widely used inhibitor of SRs, was initially employed to block the activation of SRs. Surprisingly, fucoidan failed to suppress the production of superoxide induced by HD (Fig. 5a). Quantitative analysis supported the immunofluorescence observation (Fig. 5b). Consistently, a similar level of p47^phox^ in the membrane fractions of HD-treated microglia with or without fucoidan was observed (Fig. 5c), suggesting that SRs are not required for HD-induced NOX2 activation.

**Fig. 6:**
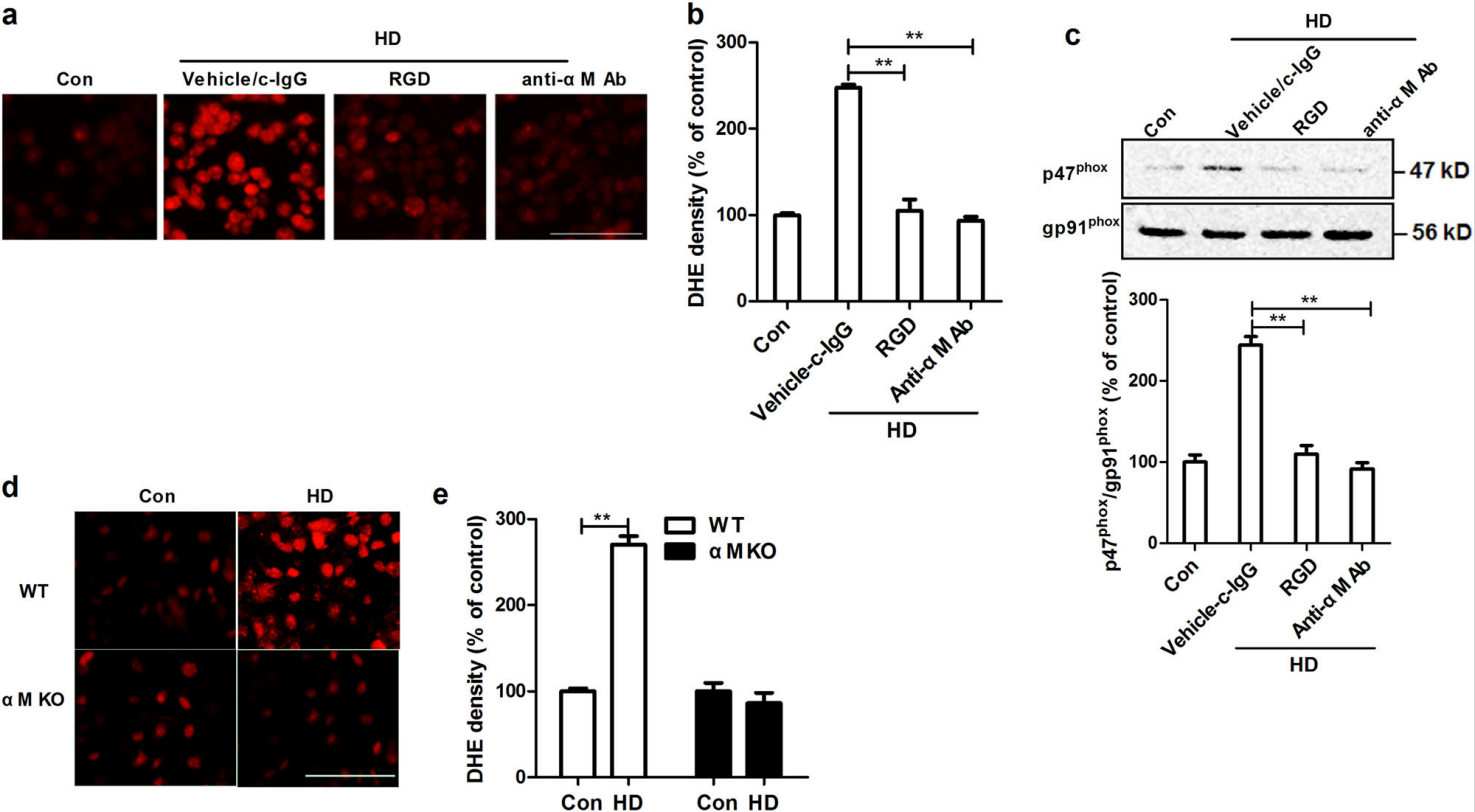
The integrin α_M_β_2_ is essential for NOX2 activation in response to HD **a** BV2 microglial cells were pre-treated with RGD or anti-α_M_ antibody and then HD-induced superoxide production was measured. c-IgG: control IgG. **b** The density of red fluorescence of DHE oxidation was quantified. **c** The effects of RGD or anti-α_M_ antibody on HD-induced membrane translocation of p47^phox^ was detected using Western blot and the density of blots was quantified. **d**, **e** HD-induced superoxide production was measured in primary mixed-glia cultures prepared from wild type and α_M_ KO mice and the density of red fluorescence of DHE oxidation was quantified. Results were expressed as a percentage of controls from three experiments performed in duplicate. *p < 0.05, **p < 0.01; *n* = 3–6; Scale bar = 100 μm

Next, we investigated whether integrin α_M_β_2_ is involved in HD-induced NOX2 activation. Integrin α_M_β_2_ activation was blocked by RGD, a peptide integrin inhibitor. Interestingly, RGD significantly reduced HD-induced NOX2 activation by showing a reduced superoxide production (red fluorescence) and membrane translocation of p47^phox^ (Fig. 6a–c). Since RGD is lack of specificity towards α_M_β_2_, the activation of α_M_β_2_ was further blocked by using antibody against α_M_. In agreement with RGD, anti-α_M_ antibody markedly decreased HD-induced superoxide production and p47^phox^ membrane translocation (Fig. 6a–c). No significant difference of microglial vitality, superoxide production, and p47^phox^ membrane translocation was observed among anti-α_M_ antibody alone, c-IgG alone, and vehicle control groups (data not shown).

To further confirm the role of α_M_β_2_ in HD-induced NOX2 activation, primary mixed-glia cultures were prepared from wild type and α_M_β_2_-deficient (α_M_^-/-^) mice. In agreement with both RGD and anti-α_M_ antibody, α_M_ deficiency reduced HD-induced superoxide production (Fig. 6d).

### The activation of Src and Erk contributes to HD-induced activation of NOX2

**Fig. 7:**
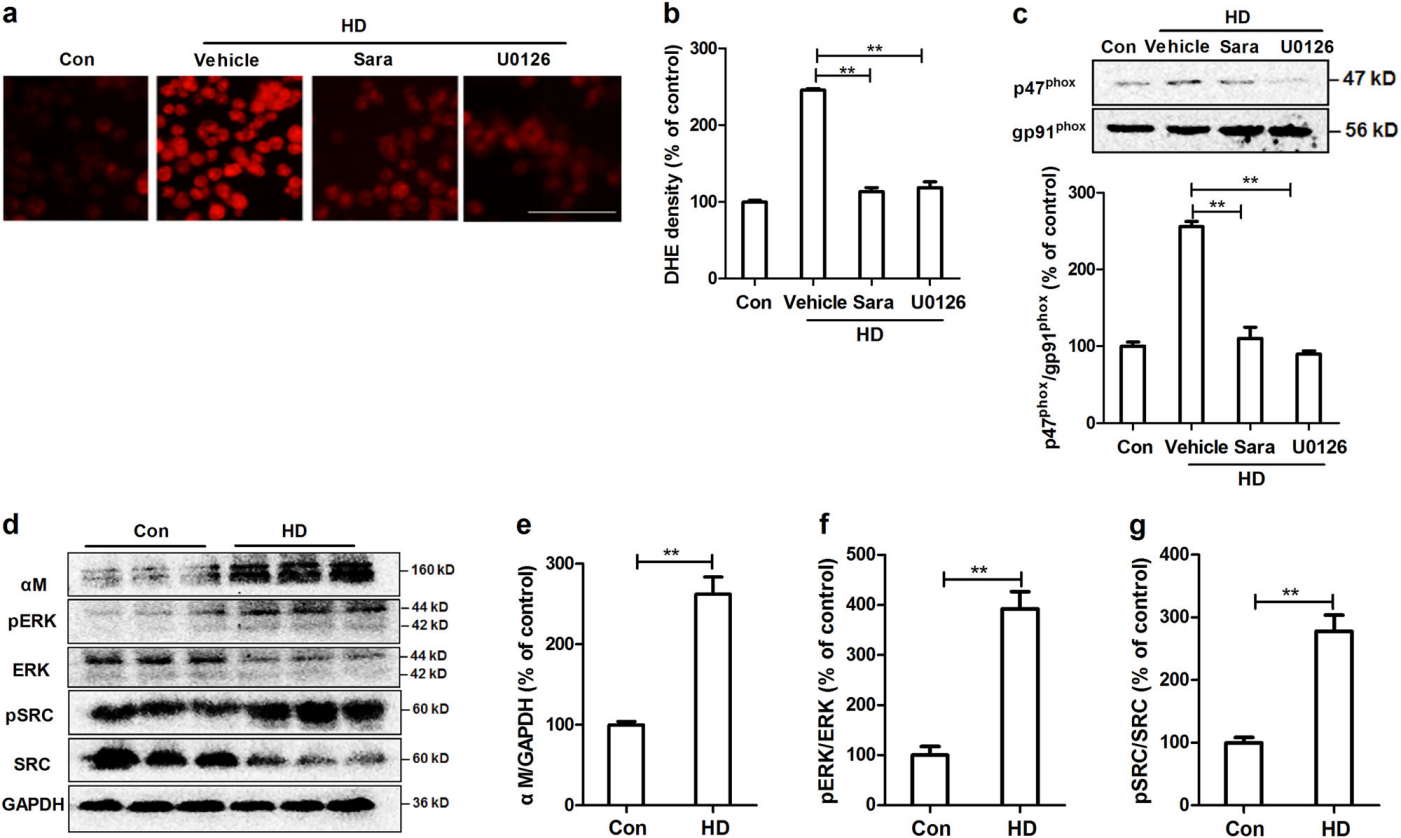
The activation of Src-Erk signals contributes to HD-induced NOX2 activation in vitro and in vivo **a** BV2 microglia were pre-treated with saracatinib (Src inhibitor) and U0126 (Erk inhibitor) prior to HD and then the production of superoxide was measured using DHE. **b** The density of red fluorescence of DHE oxidation was quantified. **c** The effects of saracatinib and U0126 on HD-induced p47^phox^ membrane translocation was detected using Western blot and the density of blots was quantified. Results were expressed as a percentage of controls from three experiments performed in duplicate. **d** The levels of α_M_, phosphorylated and total Src and Erk were determined in the midbrain tissues of HD-treated rats by Western blot and the representative blots were shown. **e**–**g** The density of blots was quantified. *p < 0.05, **p < 0.01; *n* = 3–6; Scale bar = 100 μm

The kinase of Src family is well-known to be the downstream target of integrin, in which Src is a key member^29^. Previous study revealed that Src is able to activate Erk, a kinase that can phosphorylate p47^phox^ and then induce its membrane translocation^30,31^. To investigate the role of Src and Erk in HD-induced NOX2 activation, microglial cells were pre-treated with their inhibitors, saracatinib and U0126, respectively. As seen in Fig. 7a–c, both saracatinib and U0126 prevented HD-induced superoxide production and p47^phox^ membrane translocation, indicating the activation of Src and Erk contributes to HD-induced NOX2 activation.

To determine whether α_M_β_2_-Src-Erk pathway is also involved in HD-induced NOX2 activation in vivo, the effects of HD on expression of α_M_ and activation of Src and Erk were detected in midbrain tissues of rats. Western blot analysis revealed that HD intoxication significantly elevated the expression of α_M_ in midbrain tissues of rats (Fig. 7d and e). The activation of Src and Erk was also detected in HD-treated rats by showing an increased phosphorylation of Src and Erk, compared with vehicle controls (Fig. 7d, f and g).

### Pharmacological inhibition of αMβ2-NOX2 axis attenuates HD-induced microglial activation and dopaminergic neurodegeneration

**Fig. 8:**
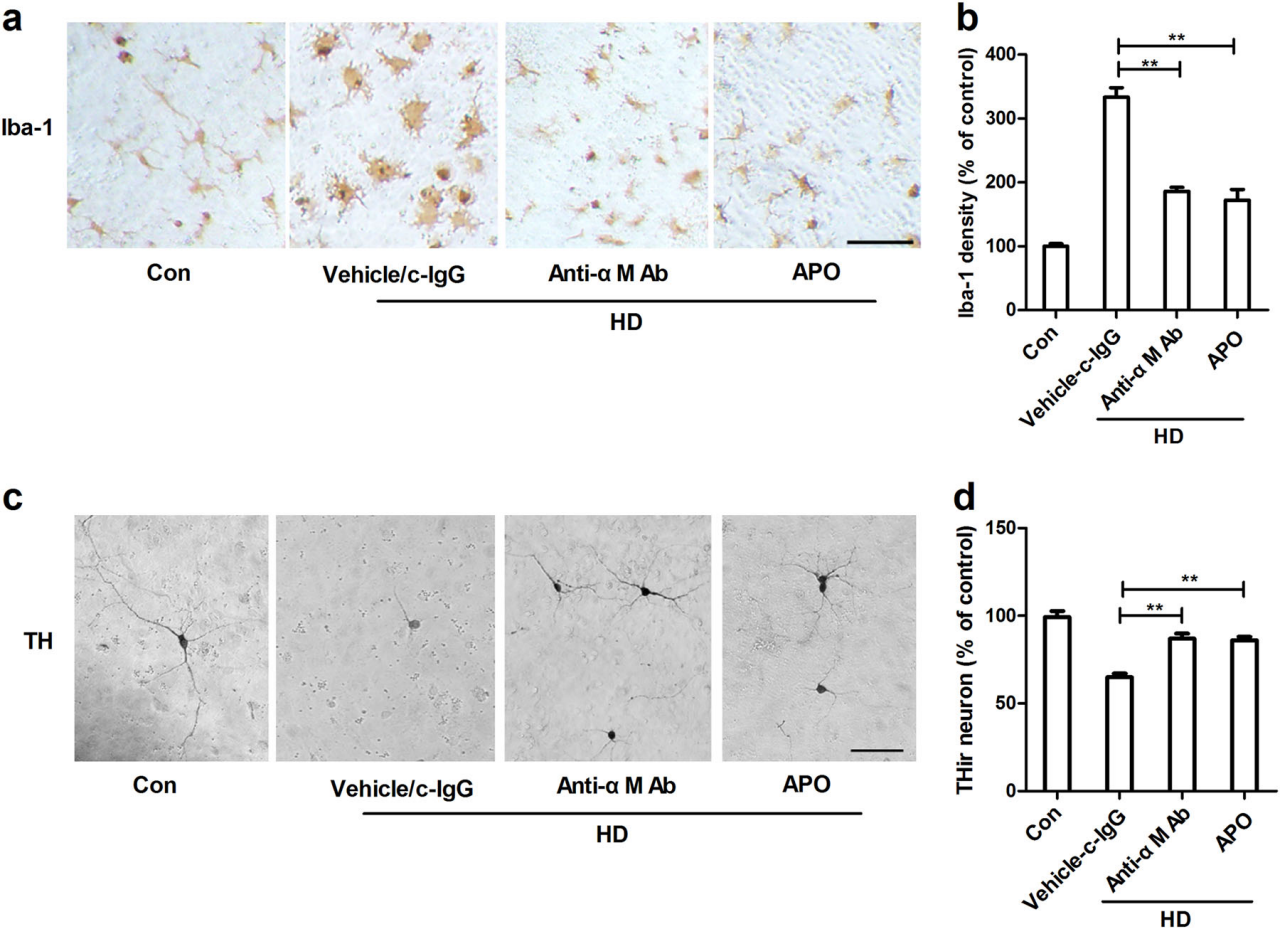
Pharmacological inhibition of α_M_β_2_-NOX2 axis attenuates HD-induced microglial activation and dopaminergic neurodegeneration **a** Rat midbrain neuron–glia cultures were pretreated with anti-α_M_ antibody or apocynin (APO) and then stimulated by HD. Microglial cells were stained with antibody against Iba-1 and the representative images were shown. c-IgG: control IgG. **b** The density of Iba-1 immunostaining was quantified. **c** Dopaminergic neurons were stained with antibody against TH and the representative images were shown. **d** The effects of anti-α_M_ antibody or apocynin on HD-induced dopaminergic neurodegeneration were assessed by THir neuron counts. Results were expressed as a percentage of controls from three experiments performed in duplicate. *p < 0.05, **p < 0.01; Scale bar = 50 μm

To investigate whether α_M_β_2_-NOX2 axis is involved in microglial activation and dopaminergic neurodegeneration induced by HD, primary midbrain neuron–glia cultures were prepared and were treated with anti-α_M_ antibody or apocynin prior to HD intoxication. Figure 8 depicted microglial activation in response to HD intoxication when compared to vehicle controls. Activated microglia cells displayed irregular morphology and intensified Iba-1 staining. By contrast, microglial activation was not observed in HD-treated cultures in the presence of anti-α_M_ antibody or apocynin (Fig. 8a and b). Importantly, inhibition of α_M_β_2_-NOX2 axis abolished HD-induced dopaminergic neurotoxicity (Fig. 8c). Analysis of THir neuron number revealed 22.17% and 19.33% protection of dopaminergic neurons by anti-α_M_ antibody and apocynin, respectively, compared with HD alone group (Fig. 8d). No significant difference of THir neuron number was observed among anti-α_M_ antibody alone, c-IgG alone and vehicle control groups (data not shown).

## Discussion

This study demonstrated that *n*-hexane/HD exposure damaged nigrostriatal dopaminergic system through α_M_β_2_-NOX2 axis-mediated microglial activation. The salient features of our study are: (1) *n*-hexane exposure resulted in loss of dopaminergic neurons in rats; (2) microglial activation preceded dopaminergic neurodegeneration and depletion of microglia reduced HD-induced dopaminergic neurotoxicity; (3) HD exposure stimulated activation of NOX2 by inducing membrane translocation of cytosolic subunit p47^phox^; (4) integrin α_M_β_2_ contributed to HD-induced NOX2 activation through a Src-Erk-dependent pathway; and (5) pharmacological inhibition of α_M_β_2_-NOX2 axis protected dopaminergic neurons against HD-induced toxicity.

Environmental toxicants play important roles in the development of sporadic form of PD. Pesticides and solvents, such as 1-methyl-4-phenyl-1,2,3,6-tetrahydropyridine (MPTP), rotenone, paraquat, and trichloroethylene, initiate a cascade of events including mitochondrial dysfunction, inflammation, and oxidative stress in the cell body that induces dopaminergic neurodegeneration and ultimately causes Parkinsonism^32–34^. *n*-Hexane is a volatile organic solvent and is widely used in industry. Previous report indicated that the concentration of *n*-hexane present in the atmosphere in the workplace is up to 879 mg/m^3^ (median values between 101 and 123 mg/m^3^)^35^. In the organism, *n-*hexane undergoes complex biotransformation, leading to the production of HD. Although there is no report for the concentration of HD in brain after *n*-hexane exposure, a positive correlation between produced HD and environmental exposure to *n*-hexane is observed^13,35,36^. In this study, by using a biologically relevant concentration, we found that HD exposure resulted in gradual loss of dopaminergic neurons in the nigrostriatal system of rats. Consistent with our finding, a previous study showed a reduced level of dopamine in the striatum of mice treated with 400 mg/kg HD for 3 weeks^18^. In addition to damage of dopaminergic neurons, HD-treated rats also displayed gait abnormality, one of the cardinal symptoms observed in PD patients^37^. These observations provide a possible explanation for the case of parkinsonism appeared in human with *n*-hexane exposure^11^.

Although the exact cause for PD has not been fully elucidated, neuroinflammation mediated by microglia has been increasingly associated with the pathogenesis of PD^38,39^. Microglia are the innate immune cells in the brain. As the first line of immune defense, resident microglia change to an activated phenotype in the conditions of brain injury or disease^40^. In addition to morphological change, activated microglia also produce a wide array of cytotoxic factors, including TNF-α, interleukin-1β, NO, and superoxide, which works in concert together to induce neuronal damage^41,42^. As early as 1988, McGeer et al. detected activated microglia, along with Lewy bodies in the SN in PD patients^43^. Microglial activation is also observed in various experimental models of PD, which has been implicated in the selective degeneration of nigrostriatal dopaminergic neurons^44,45^. We recently reported that the distribution of microglia in the SN is far greater than in the surrounding regions, contributing to the increased susceptibility of nigral dopaminergic neurons in PD^46^. In this study, microglial activation was observed in the SN of rats exposed to HD, which preceded neurodegeneration of dopaminergic neurons. We further found that the degeneration of dopaminergic neurons induced by HD was markedly reduced by removing microglia from neuron–glia cultures. These results strongly suggest that microglial activation is involved in HD-induced dopaminergic neurodegeneration.

Mechanistically, the most critical question to address is how HD activates microglia. We previously reported that NOX2 is critical for chronic microglial activation. The production of NOX2-derived superoxide is the early event during microglial activation^47^. A variety of PD-related toxicants, such as rotenone, paraquat, and the fungicide maneb, can stimulate the release of NOX2-generated superoxide, which is associated with microglial activation and dopaminergic neurodegeneration. Moreover, pharmacological inhibition or genetic deletion of NOX2 reduced microglia-mediated neurotoxicity^48^. Consistent with these reports, HD stimulated activation of NOX2 by inducing the membrane translocation of NOX2 cytosolic subunits. Moreover, inhibition of NOX2 suppressed microglial activation in response to HD, suggesting that NOX2 might be a key mediator in HD-induced microglial activation.

Next, we investigated the mechanisms behind NOX2 activation. SRs and integrin α_M_β_2_ belong to PRRs that are highly expressed in phagocytes, including microglia. Both SRs and α_M_β_2_ can identify external substances, and elicit immune responses, as well as ROS production^48^. Magwenzi et al. reported that NOX2 activation induced by oxidized low-density lipoprotein is blocked by inhibitors of CD36 (one of the most extensively studied SRs), mimicked by CD36-specific oxidized phospholipids, and ablated in CD36^-/-^ murine platelets^49^. Similarly, a SRs-dependent activation of NOX2 was also observed in microglia treated with β-amyloid, the main component of senile plaques in Alzheimer’s disease^50^. By contrast, NOX2 activation induced by LPS^51^, high-mobility group box 1^52^, diesel exhaust particles, and ultrafine carbon particles is α_M_β_2_-dependent^53,54^. Here, we provided direct experimental evidence implicating that α_M_β_2_, but not SRs, was involved in HD-induced NOX2 activation. This conclusion is supported by the following experimental findings: first, SRs inhibitor fucoidan failed to interfere with superoxide production and p47^phox^ membrane translocation in HD-treated microglia (Fig. 5); second, HD intoxication increased the expression of α_M_ (Fig. 7); third, inhibition of α_M_β_2_ attenuated activation of NOX2 induced by HD (Fig. 6); fourth, microglia deficient in α_M_β_2_ were more resistant to HD-induced NOX2 activation than wild type controls (Fig. 6); and fifth, inactivation of NOX2 by blocking α_M_β_2_ was associated with suppression of microglial activation and dopaminergic neurodegeneration in HD-treated primary cultures (Fig. 8).

Integrin α_M_β_2_ is a heterodimeric cell surface receptor and is composed of two chains, i.e., α_M_ (CD11b) and β_2_ (CD18). The activation of α_M_β_2_ stimulated by binding of extracellular ligands is capable of transmitting signals across the plasma membrane, such as changes in tyrosine phosphorylation and cytoskeletal organization^55^. The activation of tyrosine kinases, particularly Src family, is the early biochemical event. Src, one of the most studied members of Src kinase family, is rapidly activated in lymphocytes, myeloid cells, and platelets following β_2_ integrin engagement^56^. One of the essential role of Src is to phosphorylate a variety of substrates, including Erk^57–59^. Erk has been found to be able to phosphorylate p47^phox^ and subsequently induce its membrane translocation and thus NOX2 activation^60^. In this study, we found that HD intoxication elevated the phosphorylation of both Src and Erk in the midbrain of rats. Moreover, inhibition of Src and Erk mitigated HD-induced superoxide production and p47^phox^ membrane translocation, disclosing that Src and Erk signaling couples the event of HD/α_M_β_2_-induced NOX2 activation.

In summary, we found, for the first time, that *n*-hexane/HD exposure was toxic to dopaminergic neurons. Microglial α_M_β_2_-NOX2 axis was identified as a key factor in mediating HD-induced dopaminergic neurodegeneration. Our study adds to the candidate occupational/environmental triggers of PD and provides experimental evidence to validate the causal effect of toxic exposure. Due to wide usage of *n*-hexane in various industrial processes, *n*-hexane-related neurotoxicity gradually becomes a major health concern and occupational health hazard in exposed human. Early recognition of manifestations of *n*-hexane dopaminergic neurotoxicity is essential to protect workers of such professions from developing PD.

## Materials and methods

### Animal treatment

Forty adult male SD rats (9 weeks old) were purchased from the Experimental Animal Center of Dalian Medical University. Rats were housed in polycarbonate boxes, and drinking water and a commercial animal feed were available ad libitum. The animal room was maintained at ~22 °C and 50% relative humidity with a 12-h light–dark cycle. HD (Vetec™ reagent grade, 98%, Sigma, St. Louis, MO, USA) at dosage of 400 mg/kg/day was administrated to rats by intraperitoneal injection (i.p., five times per week for consecutive 5 weeks). The dosage of HD was chosen based on our previous reports^37^. The corresponding control group rats received an equivalent volume of 0.9% saline. After 1, 3, and 5 weeks of initial HD intoxication, rats (*n* = 10 each group) were sacrificed and brains were dissected. All animal procedures and their care were carried out in accordance the National Institute of Health Guide for the Care and Use of Laboratory Animals and were approved by the Institutional Animal Care and Use Committee of Dalian Medical University.

### Immunohistochemistry

For immunohistochemistry analyses, rats were perfused with PBS and followed by 4% paraformaldehyde. After perfusion, rats were decapitated, and brains were carefully removed. Brains were fixed in 4% paraformaldehyde and processed for immune-staining as described previously^21^. The primary antibodies used for immunostaining were anti-tyrosine hydroxylase (TH, EMD Millipore Corporation, Billerica, MA, USA), and anti-ionized calcium-binding adaptor molecule-1 (Iba1, Wako Chemicals, Richmond, VA, USA). The brains were sectioned into coronal slices (30 μm) with every sixth section within the SN selected for immunostaining. Formaldehyde-fixed brain slices were treated with 1% hydrogen peroxide followed by sequential incubation with blocking solution, primary and biotinylated secondary antibodies and ABC reagents. Slices were then visualized by incubating 3,3′-diaminobenzidine (DAB) according to manufacturer’s instruction.

The total number of TH-positive SNpc neurons was counted by using the optical fractionator method as described previously^61^. TH-immunoreactive neurons were counted by two individuals blind to the treatment.

### Primary cultures

Mesencephalic neuron–glia, microglia-depleted, and mixed-glia cultures were prepared according to a previously published protocol^62^.

### BV2 microglial cells

The immortalized murine microglia cell line, BV2 cells were maintained in Dulbecco’s modified Eagles medium that contained 10% fetal bovine serum and antibiotics at 37 °C in a humidified atmosphere of 5% CO_2_ and 95% air.

### Cell viability assay

The viability of BV2 microglial cells in response to HD was determined by using a commercial lactate dehydrogenase (LDH) assay kit (Beyotime, Jiangsu, China). Briefly, BV2 microglial cells were seeded at a density of 1 × 10^4^/well in a 96-well plate. After treatment with HD for 1 h, the plate was centrifuged at 400 × *g* for 5 min. Supernatant was collected and mixed with 150 μl of LDH release agents for 1 h. The plate was then centrifuged again at 400 × *g* for 5 min. Supernatant (120 μl) was collected and mixed with 60 μl LDH measurement solution in a 96-well plate. The absorbance was measured at 490 nm by using a microplate reader and the cell viability for each sample was quantified.

### Analysis for intracellular superoxide

The fluorescent marker dihydroethidium (DHE) was used to measure the generation of superoxide. Briefly, BV2 microglia or primary microglia cells were treated with HD for 24 h with or without pre-incubation of apocynin, RGD, anti-α_M_ antibody, U0126 or saracatinib for 30 min and then were loaded with DHE (10 μM). After treatment for additional 30 min, cells were rinsed twice with ice-cold PBS. Then the cells were detected for superoxide generation via fluorescence microscopy (excitation 534 nm; emission 580 nm).

### Immunocytochemistry

Immunostaining was performed as described previously^27^. Briefly, formaldehyde-fixed neuron–glia or microglia-depleted cultures were treated with 1% hydrogen peroxide followed by sequential incubation with blocking solution, primary and biotinylated secondary antibodies, and ABC reagents. Color was then developed with DAB and images were recorded. For visual enumeration of the immunostained cells in cultures, THir and Iba1ir cells in 10 representative areas per well were measured under microscope at 20× magnification.

### RNA extraction and reverse transcription-PCR

Total RNA was extracted by using RNAiso Plus according to the manufacturer’s instruction (Takara, Japan) and then quantified with a spectrophotometer. Only RNA samples with an A260/A280 of 1.8–2.2 were employed for reverse transcription. One microgram of total RNA was reverse transcribed using a reverse transcription kit (Takara, Japan). Quantitative real-time PCR was performed with a SYBR Green PCR kit (Takara, Japan) using the TP800 Real-Time PCR Detection System (Takara, Japan). The following primers, TNF-α (F: GACCCTCACACTCAGATCATCTTCT; R: CCTCCACTTGGTGGTTTGCT), iNOS (F: CTGCCCCCCTGCTCACTC; R: TGGGAGGGGTCGTAATGTCC), and GAPDH (F: TTCAACGGCACAGTCAAGGC; R: GACTCCACGACATACTCAGCACC) were used. The reaction conditions were as follows: initial denaturation at 95 °C for 5 min, followed by 40 cycles of 95 °C for 30 s, 55 °C for 30 s, and 72 °C for 30 s. The data were analyzed using the ^2−△△^CT method. GAPDH was employed as an internal control.

### Western blots analysis

Total cytosolic and plasma membrane proteins were prepared as described previously^27^. Equal amounts of protein were separated by 4–12% b-tris-polyacrylamide electrophoresis gel and transferred to polyvinylidene difluoride membranes. The membranes were incubated with primary antibody against 4-hydroxy-2-nonenal (4-HNE), α_M_, phospho-ERK1/2, ERK1/2, phospho-Src, Src (Cell Signaling Technology, Danvers, MA, USA), p47^phox^ (1:1,000; EMD Millipore, Temecula, CA, USA), gp91^phox^ (1:1,000; BD Transduction Laboratories, San Jose, CA, USA), and GAPDH (1:1000, Abcam, Cambridge, MA, USA) overnight at 4 °C and followed by horseradish peroxidase-linked anti-rabbit IgG (1:3000) for 2 h at 25 °C. ECL reagents (Biological Industries, Cromwell, CT, USA) were used as a detection system.

### Statistical analysis

Data are presented as the mean ± SEM. All data were analyzed with SPSS 11.0 for Windows. Differences in mean values between groups were tested via *t*-test or one-way ANOVA. When a significant difference was observed, pair-wise comparisons between means were analysed by LSD test. *P*-values of less than 0.05 were considered significant.
