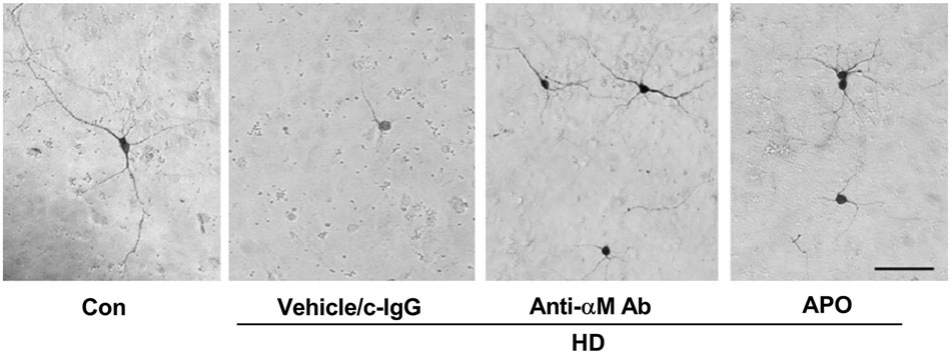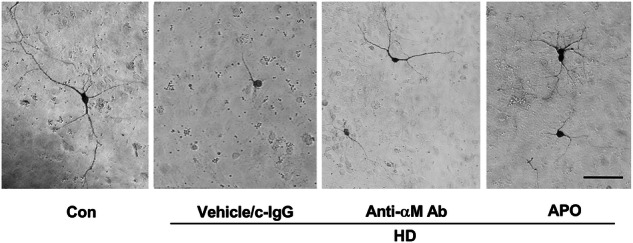# Correction: 2,5-Hexanedione induces dopaminergic neurodegeneration through integrin α_M_β2/NADPH oxidase axis-mediated microglial activation

**DOI:** 10.1038/s41419-022-05493-2

**Published:** 2022-12-13

**Authors:** Cong Zhang, Liyan Hou, Jie Yang, Yuning Che, Fuqiang Sun, Huihua Li, Qingshan Wang

**Affiliations:** 1 School of Public Health, Dalian Medical University, No. 9W. Lvshun South Road, Dalian, 116044 China; 2 Department of Cardiology, Institute of Cardiovascular Diseases, First Affiliated Hospital of Dalian Medical University, Dalian, China

Correction to: *Cell Death and Disease* 10.1038/s41419-017-0091-7, published online 19 January 2018

The original version of this article contained errors in Figures 6a and 7a. The correct figures can be found below. The authors apologize for the error.
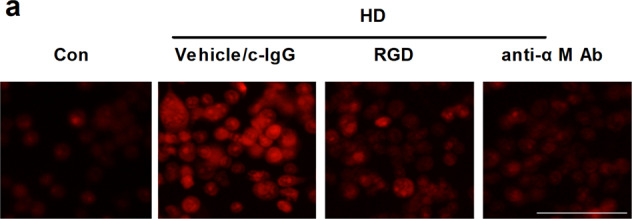

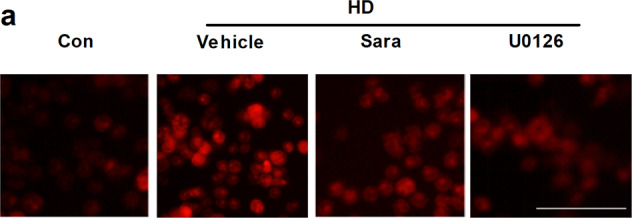


The original version of this article contained an error in Figure 8c. The correct figure can be found below. The authors assure that this correction will not affect the conclusion of this study. The authors apologize for the error.